# Accounting for blood attenuation in intravascular near-infrared fluorescence-ultrasound imaging using a fluorophore-coated guidewire

**DOI:** 10.1117/1.JBO.28.4.046001

**Published:** 2023-04-05

**Authors:** Philipp Rauschendorfer, Georg Wissmeyer, Farouc A. Jaffer, Dimitris Gorpas, Vasilis Ntziachristos

**Affiliations:** a Technical University of Munich, Chair of Biological Imaging at the Central Institute for Translational Cancer Research (TranslaTUM), School of Medicine, Munich, Germany; b Institute of Biological and Medical Imaging, Helmholtz Zentrum München, Neuherberg, Germany; c Massachusetts General Hospital, Cardiovascular Research Center, Cardiology Division, Boston, Massachusetts, United States; d Massachusetts General Hospital, Wellman Center for Photomedicine, Boston, Massachusetts, United States; e DZHK (German Centre for Cardiovascular Research), partner site Munich Heart Alliance, Munich, Germany

**Keywords:** intravascular imaging, near-infrared fluorescence imaging, intravascular ultrasound, quantification, light attenuation

## Abstract

**Significance:**

Intravascular near-infrared fluorescence (NIRF) imaging aims to improve the inspection of vascular pathology using fluorescent agents with specificity to vascular disease biomarkers. The method has been developed to operate in tandem with an anatomical modality, such as intravascular ultrasound (IVUS), and complements anatomical readings with pathophysiological contrast, enhancing the information obtained from the hybrid examination.

**Aim:**

However, attenuation of NIRF signals by blood challenges NIRF quantification. We propose a new method for attenuation correction in NIRF intravascular imaging based on a fluorophore-coated guidewire that is used as a reference for the fluorescence measurement and provides a real-time measurement of blood attenuation during the NIRF examination.

**Approach:**

We examine the performance of the method in a porcine coronary artery *ex vivo* and phantoms using a 3.2F NIRF-IVUS catheter.

**Results:**

We demonstrate marked improvement over uncorrected signals of up to 4.5-fold and errors of <11% for target signals acquired at distances up to 1 mm from the catheter system employed.

**Conclusions:**

The method offers a potential means of improving the accuracy of intravascular NIRF imaging under *in vivo* conditions.

## 1 Introduction

Intravascular ultrasound (IVUS) and intravascular optical coherence tomography (IVOCT) are imaging modalities clinically applied for inspecting the vascular system and assessing different vascular conditions, such as plaque build-up, typically as part of interventional cardiology procedures.^1^^,^^2^ These imaging techniques assess atheroma burden based on anatomical landmarks, such as vessel stenosis or thinning of the fibrous cap, but fail to visualize key pathophysiological processes implicated in atheroma formation and rupture risk, such as inflammation or endothelial permeability.^1^^–^^3^ Near-infrared fluorescence (NIRF) imaging has been developed to operate in tandem with IVUS or IVOCT and provide a more comprehensive inspection of the vascular lumen. The method employs fluorescent agents that can outline biological processes *in vivo*, enabling visualization of altered pathophysiology.^1^^,^^3^ Hybrid NIRF–IVUS and NIRF–IVOCT has been applied in animal^4^^,^^5^ and human^6^ intravascular studies to reveal arterial/plaque inflammation, stent-related inflammation, atheroma-associated increased permeability, fibrin-deposition, thrombosis, and other conditions.^4^^,^^5^^,^^7^

Successful application of intravascular NIRF imaging considers operation through blood, especially when combined with IVUS imaging. One advantageous feature of IVUS over IVOCT is that it does not require flushing with a contrast medium for blood clearing in the vessel imaged to facilitate image formation. By operating in the near-infrared spectral region, NIRF imaging capitalizes on the low light attenuation in tissue in this spectral range to also operate through blood. However, as the distance between the NIRF detector and the vessel wall changes during intravascular imaging pullbacks, this attenuation varies, leading to corresponding fluorescence signal variation. Accurate quantification, therefore, requires methods that can take into account the blood attenuation at the wavelength of operation and the distance between the NIRF detector and the vessel wall that contains the fluorescence source.

The parallel operation of NIRF and IVUS allows for accurate coregistration of the two modalities, a feature that has been previously explored to measure the distance between the NIRF detector and the vessel wall on the ultrasound images.^2^^,^^4^^,^^8^^–^^11^ This distance is then utilized in a correction model for each point measured on the vessel wall. Previous works assumed blood attenuation using *ex vivo* reference measurements or approximate average values estimated *in vivo*.^4^^,^^9^^–^^11^ However, such approximations can introduce errors since they do not account for deviations of each patient from the average value employed in the correction model; for example, due to variations of hematocrit,^8^ oxygenation state,^12^ or fluctuations in blood flow^13^^,^^14^ as a function of vessel type, intravascular location, and pathological state.^15^

We introduce a method to accurately determine NIRF signal attenuation by blood during the examination, offering an adaptive correction scheme tailored not only to each patient but also to each imaging frame collected during the intravascular procedure. In particular, we postulated that a fluorescent coating applied to the guidewire used to steer the NIRF–IVUS catheter could provide a reference signal for accurate attenuation measurements during intravascular imaging. The underlying premise is that collection of attenuated NIRF signals from the fluorophore-coated guidewire can be employed to compute the light attenuation due to blood, as a function of distance, for each frame location, thus taking into account local and general blood attenuation characteristics. Then, combination of this position-(frame-)dependent blood attenuation with NIRF detector-to-vessel wall distance computed on the IVUS images can be employed to offer an accurate correction scheme for intravascular NIRF imaging. Intravascular imaging methods such as IVUS/IVOCT and optoacoustic have been employed to sense parameters, such as blood flow^16^^–^^19^ and hematocrit,^8^ and hence could be used to estimate aspects of blood attenuation dynamically. However, to achieve accurate correlation to light attenuation, the required number of such readouts, which have to be laboriously compiled into an extensive look-up-table, is unknown at present. In contrast, our method offers the means to quantify the attenuative effect blood has on NIRF signals directly. The work was motivated by the recent development of biocompatible fluorescence coatings^20^^,^^21^ that could lead to a viable solution for the clinical application of such a correction scheme.

To examine this premise and the performance of the new method, we first performed *ex vivo* measurements of a blood-perfused porcine coronary artery injected with indocyanine green (ICG). A fluorophore(ICG)-coated guidewire, placed inside of the artery lumen, guided a 3.2F NIRF–IVUS imaging catheter, i.e., a catheter of dimensions appropriate for coronary intravascular imaging. Measurements from the fluorophore-coated guidewire were employed to examine the performance of the of fluorescent signals attenuated by blood absorption and scattering, against control measurements performed through water. To gain further insights on the optimal performance of the method, under varying conditions of attenuation and distance, we also employ a phantom comprising slanted capillaries filled with ICG to simulate the conditions of the guidewire and tissue NIRF signals. In the following, we present the proof of principle of the method and the experimental measurements performed, showcase our results, and discuss the findings and the clinical potential of the proposed method.

## 2 Methods

### 2.1 3.2F NIRF–IVUS Imaging System

**Fig. 1:**
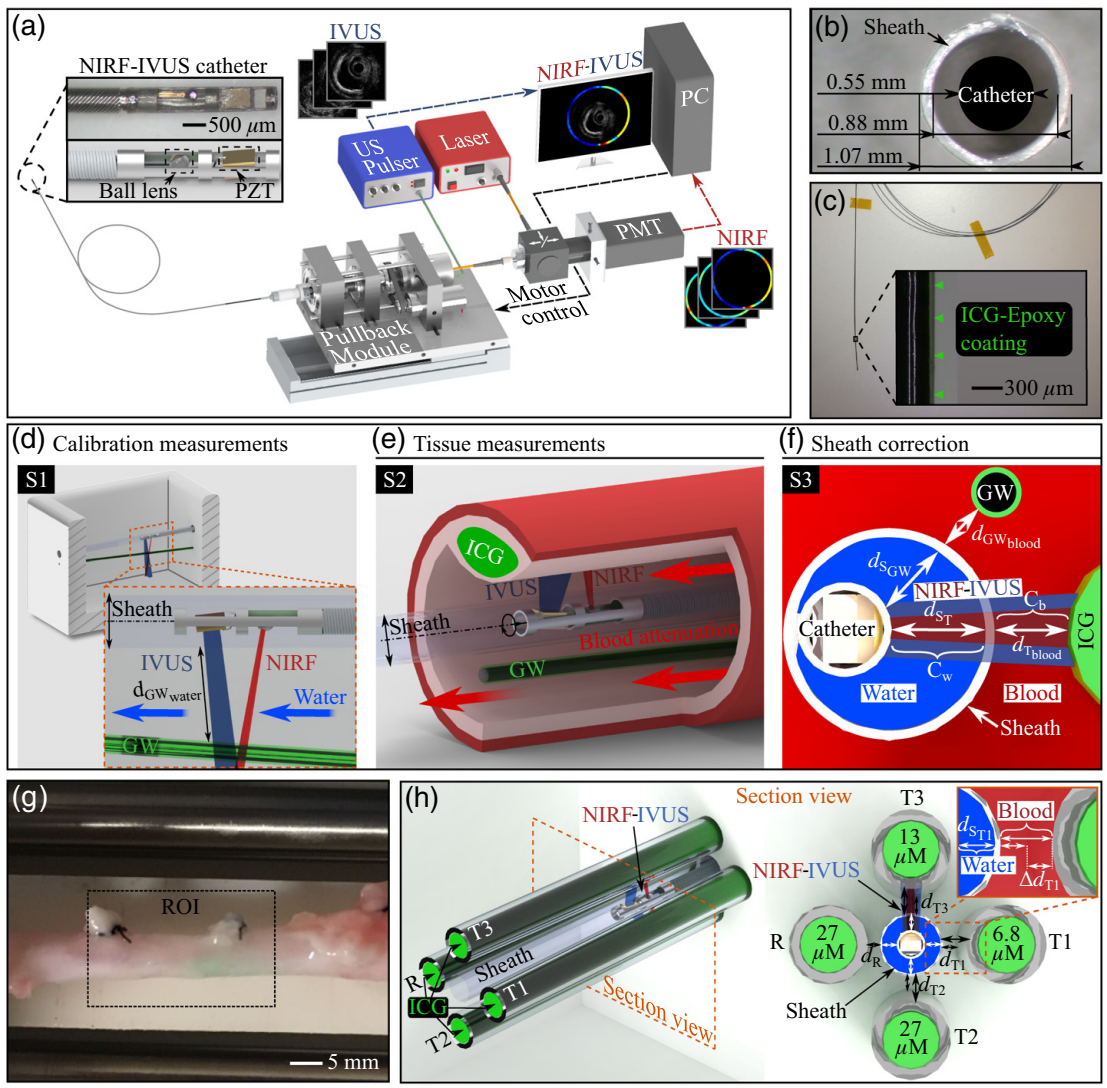
Imaging setup and description of experiments. (a) Illustration of NIRF–IVUS imaging setup including an intravascular 3.2F NIRF–IVUS catheter. (b) Cross-sectional image visualizing the dimensions of the NIRF–IVUS catheter inserted into a size 3.2F sheath. (c) Image of a commercial intravascular guidewire coated with epoxy resin and ICG. (d) Schematic of the calibration phantom used to obtain NIRF–IVUS measurements of the fluorophore-coated guidewire at variable distances (sheath-guidewire distances, $d_{{\mathrm{GW}}_{\text{water}}}$) in water to provide a correction factor for NIRF signal loss in water [$C_{w}$, Eq. (1)] and construct a look-up-table containing NIRF reference signals. (e) Schematic of an intravascular measurement of an ICG-injected artery perfused with blood, with the adjacent fluorophore-coated guidewire providing reference signals for the calculation of frame-by-frame correction factors for NIRF attenuation in blood [$C_{b}$, Eq. (4)]. (f) Cross-sectional schematic of an intravascular measurement showing the distances between the catheter and the sheath ($d_{s_{\mathrm{GW}}}$ and $d_{s_{T}}$) used to correct for signal loss in water within the sheath, see Eqs. (2) and (3), the sheath and the guidewire ($d_{{\mathrm{GW}}_{\text{blood}}}$), and the sheath and the tissue ($d_{T_{\mathrm{blood}}}$) containing ICG. (g) Image of an ICG-injected porcine coronary artery indicating ROI for imaging and correction analysis. (h) Schematic of the capillary phantom. The phantom contains three different target capillaries (T1-3) and a reference (R) capillary that simulates the fluorophore-coated guidewire, each filled with solutions of ICG at different concentrations. The target capillaries are positioned at variable distances ($d_{T1-3}$) resulting in a range ($\Delta d_{T1-3}$), whereas the reference capillary was positioned at a fixed distance ($d_{R}$) from the catheter sheath. All capillaries are exposed to signal loss in water ($C_{w}$) inside the sheath ($d_{S_{T1-3}}$, $d_{S_{R}}$) and attenuation due to blood ($C_{b}$) outside the sheath. In all panels: NIRF, near-infrared fluorescence; IVUS, intravascular ultrasound; ICG, indocyanine green; GW, guidewire; and ROI, region of interest.

The hybrid NIRF–IVUS imaging system consists of a control and readout system, a helical pullback module, and a 3.2F NIRF-IVUS imaging catheter [Fig. 1(a)]. An ultrasound pulser-receiver (5073PR, Panametrics, Waltham) was used to excite and detect IVUS signals. A 750-nm fiber-coupled laser (B&W Tek, Newark, Delaware) was implemented to excite NIRF signals, which were detected by a photomultiplier tube (Model H7422-50, Hamamatsu, Japan). The pullback module includes a fiber-optic rotary joint combined with an electrical slip ring (Alpha Slip Rings, Austin, Texas), a rotary motor (3268G042BX4AES-4096, Faulhaber, Germany), and a linear stage (X-LRQ stage, Zaber, Vancouver, BC, Canada).

The miniaturized NIRF-IVUS catheter previously developed by our group^11^ was used to implement all the measurements described herein. In short, the catheter comprises a ball-lens attached to a 50/105-μm fiber (Thorlabs, Newton, New Jersey) and a piezoelectric element (size: 0.4×0.6  mm; center frequency: 40 MHz; Blatek, Boalsburg, Pennsylvania) for NIRF-IVUS signal excitation and detection. Both sensors are implemented into a ferrule in a serial design featuring a total outer diameter of 0.55 mm. The NIRF–IVUS catheter is introduced into size 3.2F catheter sheath [Fig. 1(b)] made from low-density polyethylene and combined with a custom-made flushing port that utilizes a clinical introducer sheath (RT-R50G10PQ, Terumo Europe) coupled to the catheter sheath via three-dimensional (3D) printed flexible connector. The sheath was filled with distilled water to ensure ultrasound coupling into the tissue and prevent blood contact with the NIRF sensor. The distance between the catheter and the sheath wall must be accounted for when correcting fluorescence intensities [see Fig. 1(f) and Eqs. (2) and (3)].

All data presented herein were acquired with 60-rpm rotation speeds and 0.25-mm/s pullback speeds for a distance of 20 mm (i.e., 80 cross-sectional NIRF/IVUS imaging frames), resulting into a helical scan of the intimal arterial wall. A custom-made LabVIEW (National Instruments Corp., Austin, Texas) software allows for system control and data acquisition, whereas all data analysis was implemented in MATLAB (Mathworks, Natick, Massachusetts).

### 2.2 Guidewire Coating

A standard clinical guidewire with a 0.35-mm diameter (${\mathrm{PT}}^{2}$ Moderate Support, Boston Scientific Corporation, Marlborough, Massachusetts) was immersed in a 50-μM solution of ICG in epoxy resin and cured at room temperature. The method has been optimized to afford a thin and homogeneous fluorescent coating with an average thickness of 45  μm [Fig. 1(c)] and a high NIRF signal-to-noise ratio (SNR) based on an ICG concentration, which was maximized until quenching occurs above 50  μM.^22^

### 2.3 Calibration Measurements

The method consists of a calibration step [Fig. 1(d)], which is performed once for every guidewire prior to tissue measurements [Fig. 1(e)]. For this calibration step, the fluorophore-coated guidewire was measured through water in a phantom with varying distances (0.1 to 1. mm) between the guidewire and the catheter sheath wall to (i) calculate a correction factor for water [$C_{w}$, Eq. (1)] and (ii) construct a look-up-table containing NIRF signals for variable distances. The water correction factor was used to account for NIRF signal loss between the NIRF sensor and the sheath wall [Fig. 1(f), Eqs. (2) and (3)] and between the sheath wall and tissue to calculate ground truth values [Secs. 2.4–2.5, Eq. (6)]. The data from the look-up-table served as the reference values to calculate a correction factor for blood attenuation [$C_{b}$, Eq. (4)]. The calibration phantom comprised a 3D-printed reservoir 26×21×18  mm (L×W×H) in size with designated placeholders that allowed the guidewire to be angled relative to the imaging catheter.

### 2.4 *Ex Vivo* Tissue Measurements

**Fig. 2:**
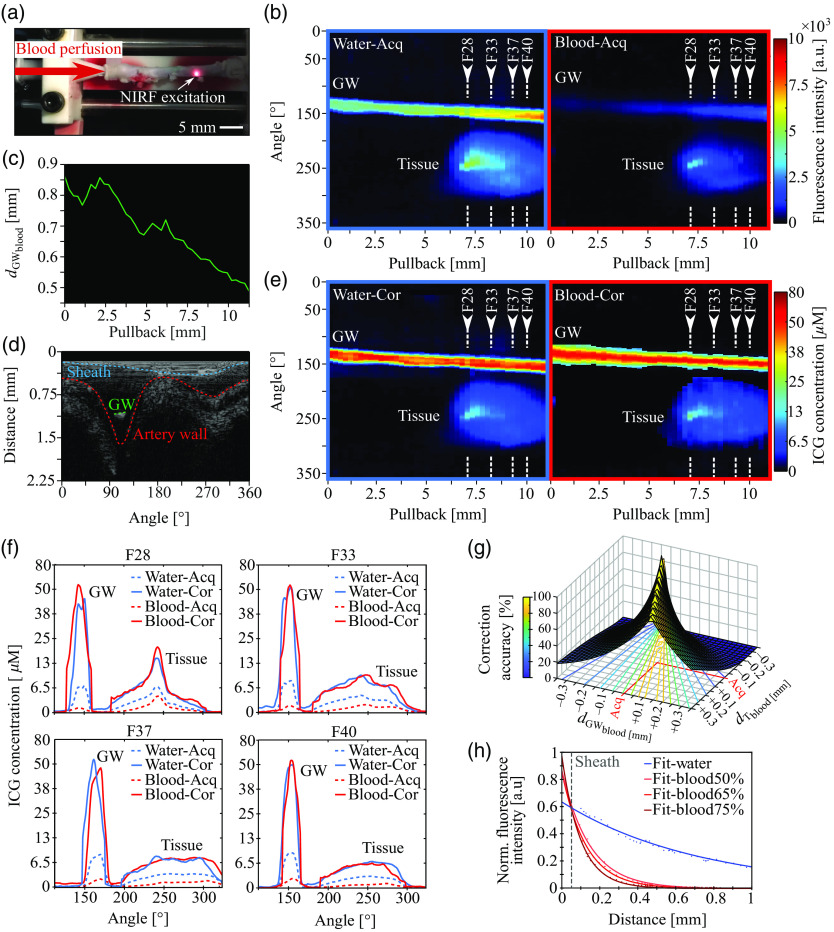
Validation of the correction method in an excised pig artery. (a) NIRF–IVUS measurements of a porcine coronary artery placed in a water bath and perfused with blood. (b) Acquired uncorrected intravascular NIRF maps of an ICG-injected porcine artery measured through water and a 75% blood solution, showing NIRF signals from both the guidewire (GW) and tissue. (c) Distance between the fluorophore-coated guidewire and the catheter sheath measured during blood perfused tissue measurements ($d_{{\mathrm{GW}}_{\text{blood}}}$) per pullback position (frame). (d) Exemplary IVUS imaging frame in polar coordinates showing distances from the sensor to the catheter sheath, the fluorophore-coated guidewire (GW), and the artery wall tissue. (e) The same intravascular NIRF maps as in (b) after correction showing quantified ICG concentrations. (f) Selected frames showing uncorrected intensity values and corresponding corrected ICG concentrations for the GW and tissue signals through blood and water. (g) Sensitivity analysis investigating the influence of simulated errors in sheath to guidewire ($d_{{\mathrm{GW}}_{\text{blood}}}$) and tissue ($d_{T_{\text{blood}}}$) distance measurements on correction accuracy acquired during blood perfused tissue measurements; (h) NIRF measurements of the fluorophore-coated guidewire imaged through water and 50%, 65%, and 75% blood mixtures with corresponding one-term exponential model (averaged NIRF intensities shown as points and fits as solid lines; $R^{2}$ for all fits = 0.99). In all panels: ICG, indocyanine green; GW, guidewire; Acq, acquired; Cor, corrected.

A fresh pig heart was collected from a butcher shop and a 40-mm segment of the right coronary artery with an inner diameter of ∼2.8  mm was excised and positioned into a designated perfusion holder [Fig. 1(g)]. An aqueous solution of ICG (38  μM) was injected into the media layer of the artery to create a localized target fluorescence signal in the tissue. The fluorophore-coated guidewire (described in Sec. 2.2) was inserted into the vessel lumen first, followed by the imaging catheter. By inserting the guidewire into an opening at the distal end of the catheter sheath, the imaging catheter was guided to its starting position for imaging next to the guidewire, similar to the configuration in standard clinical intervention procedures [see Fig. 1(e)]. Figure 1(g) shows the region-of-interest (ROI) visualizing the location of fluorophore injection and the segment for final data analysis. We performed two NIRF–IVUS pullbacks of the artery in a water bath while perfusing it once with water (as a control) and once with a solution of 75% blood diluted with an isotonic solution of aqueous 0.9% NaCl [Fig. 2(a)]. Finally, the attenuated NIRF maps were corrected frame-by-frame using the NIRF reference signals acquired from the guidewire as described in Sec. 2.5. The ICG concentrations obtained after correction of blood attenuation were then compared with the ground truth values calculated from the control measurements through water [Sec. 2.5, Eq. (6)] for each angular position in all frames to evaluate the quantification accuracy of the method. We also performed a sensitivity analysis to investigate the influence of IVUS distance measurements on correction accuracy. For this, we altered the distances between the sheath wall and the guidewire ($d_{{\mathrm{GW}}_{\text{blood}}}$) and tissue $\left(d_{T_{\text{blood}}}\right)$ in ±50  μm steps from the values acquired in a representative frame and angular location and calculated quantification accuracy for each deviation as described above.

### 2.5 Fluorescence Signal Correction

The same fluorophore (ICG) was used to coat the guidewire and injected into the tissue. The fluorophore-coated guidewire was measured concurrently with the vessel wall to enable readouts that accurately depict the true distribution of ICG in the tissue.^23^^,^^24^ Light attenuation by blood in intravascular fluorescence imaging was modeled as an exponential function of the distance between the outer wall of catheter sheath and the vessel wall or fluorophore-coated guidewire.^4^^,^^8^^–^^11^ The NIRF signals of the guidewire were segmented from tissue signals using the coregistered IVUS data.

Correction factors for blood attenuation were calculated for each frame (see details below) by comparing NIRF signals of the guidewire acquired during the tissue measurements through blood to the reference values obtained during the calibration measurements through water. Fluorophore concentrations in the tissue were calculated using these blood correction factors, and the known distances between the catheter and the artery wall (manually delineated in coregistered IVUS frames). In all calculations, NIRF signal loss in the water-filled gap between the NIRF sensor and the catheter sheath is accounted for. The steps of our correction method for a typical measurement are described in detail below (S1-S5):

(S1) We first performed an imaging pullback with the imaging catheter (Sec. 2.3) in a calibration phantom to obtain NIRF–IVUS measurements of the fluorophore-coated guidewire through water at variable distances [Fig. 1(d)]. For each cross-sectional frame generated during the imaging pullback, the mean intensity (highest five values averaged) for the guidewire was obtained from the NIRF modality ($I_{{\mathrm{GW}}_{\text{water}}}$) and the distance between the guidewire and the sheath wall ($d_{{\mathrm{GW}}_{\text{water}}}$) was calculated from the coregistered IVUS modality. Using the intensity and distance values obtained from an entire imaging pullback in the phantom, we extracted a correction factor for the signal loss due to water ($C_{w}$, ${\mathrm{mm}}^{-1}$) using an exponential fitting model [Eq. (1)]:
  $$I_{{\mathrm{GW}}_{\text{water}}}=I_{0_{{\mathrm{GW}}_{\text{water}}}}\times e^{{-C}_{w}\times d_{{\mathrm{GW}}_{\text{water}}}},$$ (1)
  where $I_{{\mathrm{GW}}_{\text{water}}}$ is the measured intensity of the guidewire in water and $I_{0_{{\mathrm{GW}}_{\text{water}}}}$ is the incident intensity determined by the model fit.
(S2) We then inserted the same catheter and guidewire from (S1) into an *ex vivo* coronary artery and performed an imaging pullback during blood perfusion [Fig. 1(e), Sec. 2.4]. For each cross-sectional frame generated during the imaging pullback, the mean intensity value for the guidewire ($I_{{\mathrm{GW}}_{\text{blood}}}$) and many intensity values for the ICG-injected tissue ($I_{T_{\text{blood}}}$) were obtained from the NIRF modality, whereas the water-filled distances between the NIRF detector and the sheath wall at the angular locations of the guidewire ($d_{S_{\mathrm{GW}}}$) and the tissue ($d_{S_{T}}$) and the blood-filled distances between the sheath wall and the guidewire ($d_{{\mathrm{GW}}_{\text{blood}}}$) and tissue ($d_{T_{\text{blood}}}$) were calculated from the coregistered IVUS modality [see Fig. 1(f)].
(S3) Before the values obtained in (S1) and (S2) could be applied to correct the tissue signals ($I_{T_{\text{blood}}}$) for attenuation by blood, we first considered the propagation of the signal through both blood ($d_{{\mathrm{GW}}_{\text{blood}}}$ and $d_{T_{\text{blood}}}$) and water ($d_{S_{\mathrm{GW}}}$ and $d_{S_{T}}$), which was accounted for by removing the water’s contributions to the signals using $C_{w}$, as shown in Eqs. (2) and (3):
  $$I_{T^{*}}=I_{T_{\text{blood}}}\times e^{(-C_{w})\times d_{S_{T}}},$$ (2)
  $$I_{{\mathrm{GW}}^{*}}=I_{{\mathrm{GW}}_{\text{blood}}}\times e^{(-C_{w})\times d_{S_{\mathrm{GW}}}},$$ (3)
  where $I_{T^{*}}$ and $I_{{\mathrm{GW}}^{*}}$ are the water-corrected NIRF intensity from the tissue and guidewire, respectively.
(S4) Next, a correction factor for blood attenuation ($C_{b}$, ${\mathrm{mm}}^{-1}$) was calculated for each frame of the artery measurement using the water-corrected intensity of the guidewire ($I_{{\mathrm{GW}}^{*}}$) and the intensity of the guidewire in the calibration measurement ($I_{{\mathrm{GW}}_{\text{water}}}$) at the same distance from the sheath ($d_{{\mathrm{GW}}_{\text{blood}}}=d_{{\mathrm{GW}}_{\text{water}}}$) while assuming equal incident intensities [$I_{0_{{\mathrm{GW}}_{\text{water}}}}=I_{0_{{\mathrm{GW}}_{\text{blood}}}}$; Eq. (4)]:
  $$C_{b}=\frac{\log(\frac{I_{{\mathrm{GW}}_{\text{Water}}}}{I_{{\mathrm{GW}}^{*}}})}{d_{{\mathrm{GW}}_{\text{water}/\text{blood}}}}{-C}_{w}.$$ (4)
(S5) Finally, the water-corrected NIRF intensities from the tissue for each frame ($I_{T^{*}}$) were corrected for the signal attenuation from blood using the respective correction factor for blood attenuation ($C_{b}$) for that frame [Eq. (5)]:
  $$I_{T^{**}}=I_{T^{*}}\times e^{C_{b}\times d_{T_{\text{blood}}}},$$ (5)
  where $I_{T^{**}}$ is the final blood-corrected NIRF intensity of the tissue and $d_{T_{\text{blood}}}$ is the distance between the sheath wall and the tissue containing the ICG. The NIRF intensities from the tissue acquired during the control measurements ($I_{T_{\text{water}}}$) were corrected for the signal loss by water using the respective correction factor for water ($C_{w}$) for that frame [Eq. (6)]:
  $$I_{\mathrm{GT}}=I_{T_{\text{water}}}\times e^{C_{w}\times (d_{T_{\text{water}}}-d_{S_{T}})},$$ (6)
  where $I_{\mathrm{GT}}$ is the ground truth NIRF intensity of the tissue and $d_{T_{\text{water}}}$ is the distance between the outer sheath wall and the tissue containing the ICG through water. The conversion of $I_{T^{**}}$ and $I_{\mathrm{GT}}$ to ICG concentrations was estimated using $I_{{\mathrm{GW}}^{*}}$ as a reference and correlating it to the ICG concentration in the guidewire coating.

### 2.6 Validation of the Method with a Capillary Phantom Experiment

The accuracy and robustness of the fluorescence signal correction for variable imaging conditions using a guidewire-like reference signal was assessed using two phantoms: a capillary-calibration (calibration measurements) and a target phantom [Fig. 1(h)]. The capillary-calibration phantom replicated the calibration measurements in Sec. 2.3 and contained only a single capillary filled with 27  μM ICG dissolved in DMSO, which was angled to create imaging distances ranging from 0.45 to 1.6 mm [similar like Fig. 1(d)]. The target phantom-simulated NIRF signals at different concentrations and distances using three glass capillaries (25  μl per capillary), which were filled with 6.8 (T1), 13 (T3), and 27 (T2) μM ICG dissolved in DMSO and angled within the phantom to create variable distances ($d_{T1-3}$) between the wall of the catheter sheath and the targets, ranging from 0.55 to 1 mm [$\Delta d_{T1-3}$, Fig. 1(h)]. A fourth capillary was filled with the same ICG concentration as in the capillary-calibration phantom (27  μM), fixed at ∼0.45  mm distance ($d_{R}$), and was used to acquire a frame-by-frame reference (R) signal. NIRF intensities in both the target and capillary-calibration phantoms were recorded via helical pullbacks similar to the tissue measurements. Recorded NIRF intensities were dependent on the distance between the sheath wall and the capillaries, as well as the distance between the sheath wall and the NIRF detector [$d_{S_{T1-3}}$ and $d_{S_{R}}$, see Fig. 1(h)] as mentioned in Sec. 2.5. While the capillary-calibration phantom was imaged through water, the target phantom was imaged through water (as a control) and solutions of blood diluted to 75% and 50% with an isotonic solution of aqueous 0.9% NaCl. The data from the capillary-calibration measurement were fit with an exponential function to model NIRF signal loss in water. Finally, we corrected the blood-attenuated intensity of the target capillaries within the target phantom using the reference signal as described in Sec. 2.5 and compared the agreement of the corrected intensities with the ground truth values calculated from the control measurements through water [similar like in Sec. 2.5, Eq. (6)] to estimate the correction accuracy. Furthermore, we correlated corrected NIRF intensities to ICG concentrations to estimate the accuracy of fluorophore quantification.

## 3 Results

### 3.1 Correcting Signal Attenuation by Blood Using a Fluorophore-Coated Guidewire as a Reference

Figure 2 shows NIRF–IVUS measurements of a fluorophore-coated guidewire inside of an ICG-injected *ex vivo* pig coronary artery (see Sec. 2.4) that was perfused alternately with blood [Fig. 2(a)] and water (as a control), recorded to assess the feasibility of correcting NIRF signals for blood attenuation on a frame-by-frame basis using the guidewire signal as a reference. Figure 2(b) shows acquired uncorrected NIRF maps of imaging pullbacks through water and 75%-blood dilution. As expected, the recorded NIRF intensities were significantly lower in the presence of blood due to increased attenuation when compared with the measurements in water. Furthermore, the fluorescent signals from the guidewire showed variable intensities throughout the pullbacks due to changing distances from the sheath [Fig. 2(c)], as quantified by IVUS [Fig. 2(d)]. The intensity distribution of the signal from the ICG in the tissue is affected by both postinjection fluorophore diffusion and variations in sheath–tissue distance. Figure 2(e) shows the corresponding NIRF maps after correction of the intensities [see Secs. 2.3–2.5 and Figs. 1(d)–1(f)] to afford ICG concentrations throughout the pullback (see colorbar). Figure 2(f) shows acquired uncorrected and corrected concentration profiles of the guidewire and the tissue from four exemplary frames [Figs. 2(b), 2(e); F28, F33, F37, and F40], to more closely evaluate quantification accuracy. The quantified concentrations based on the corrected water measurements served as the ground truth [see Sec. 2.5, Eq. (6)]. The estimated ICG concentrations in blood corresponded well to these ground truth measurements in water for both the signals from the guidewire and the tissue. After correction, the calculated ICG concentrations in the tissue measured through blood were 89% (variance = 9.6%) accurate on average compared with the measurements through water, which yielded an improvement of 2.2-fold compared with uncorrected measurements. The correction accuracy of our method was dependent on the IVUS distance measurements [see Eqs. (4) and (5) and Fig. 2(g)]. Our sensitivity analysis indicated that our method achieved an average correction accuracy of 70% for simulated errors in IVUS distance measurements between the sheath wall and the guidewire ($d_{{\mathrm{GW}}_{\text{blood}}}$) and tissue ($d_{T_{\text{blood}}}$) of up to ±100  μm.

### 3.2 Evaluating Attenuation Correction Efficacy Versus Distance and Fluorophore Concentration in a Capillary Phantom

**Fig. 3:**
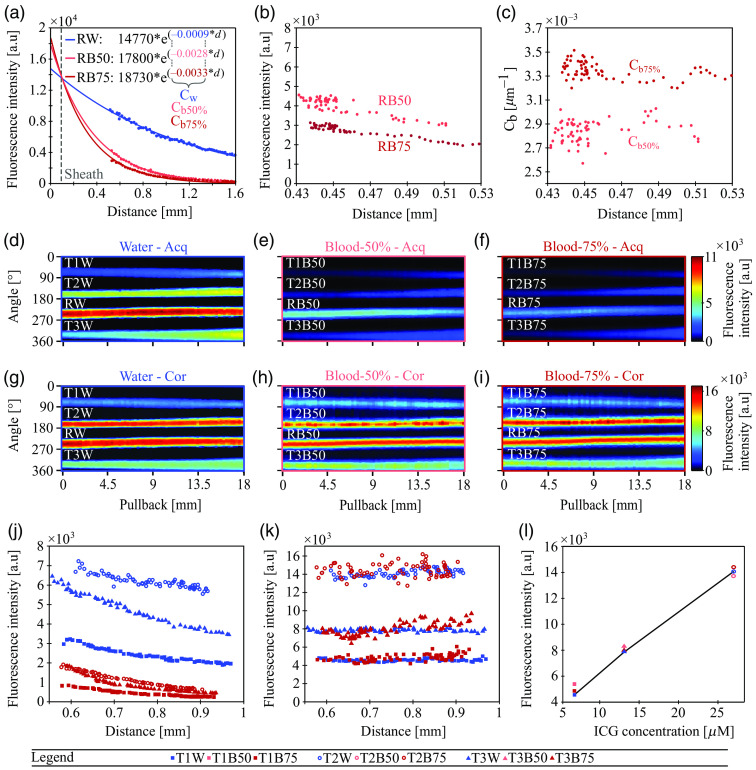
Validation of correction method for intravascular NIRF with capillary phantoms. (a) Measurements of the capillary-calibration phantom in water and both blood dilutions (for validation) showing NIRF intensities (averaged per frame) versus distance from the NIRF sensor to the reference capillary, with corresponding model fits and values for $C_{w}$ and $C_{b}$ (data points shown as dots and fits as solid lines). (b) NIRF intensities (averaged per frame) of the reference capillary recorded during target measurements in 50% blood (RB50) and 75% blood (RB75) at distances from the sheath wall to the reference capillary ranging between 0.43 and 0.53 mm. (c) Calculated correction factor ($C_{b}$) for the 50% and 75% blood mixtures versus distance from the sheath wall to the reference capillary for each frame. Acquired uncorrected NIRF maps of reference and target capillaries measured through (d) water, (e) 50% blood, and (f) 75% blood. Corrected NIRF maps of reference and target capillaries measured through (g) water, (h) 50% blood, and (i) 75% blood. (j) Acquired uncorrected NIRF intensities (averaged per frame) from the target capillaries measured in the target phantom through water (T1-3W) and 75%-blood mixture (T1-3B75) plotted versus imaging distance between the sheath wall and capillary. (k) Corrected NIRF intensities ($I_{T**}$, averaged per frame) from the target capillaries measured in the target phantom through water (T1-3W) and 75%-blood mixture (T1-3B75) plotted versus imaging distance between the sheath wall and capillary. (l) Quantification of ICG concentration via corrected NIRF intensities ($I_{T**}$, averaged over pullback) of the three different target capillaries measured through water, 50% blood, and 75% blood. RW, Reference capillary measured in water; RB50, reference capillary measured in 50% blood mixture; RB75, reference capillary measured in 75% blood mixture; $C_{w}$, correction factor for water; $C_{b50\%}$, correction factor for the 50% blood mixture; $C_{b75\%}$, correction factor for the 75% blood mixture; Acq, acquired; Cor, corrected; T1-3W, target capillaries 1 to 3 measured in water; T1-3B50, target capillaries 1 to 3 measured in 50% blood mixture; T1-3B75, target capillaries 1 to 3 measured in 75% blood mixture; ICG, indocyanine green.

Pullback measurements of the capillary-calibration phantom afforded dozens of data points (highest five intensity values averaged per frame) at distances of 0.5 to 1.6 mm from the sheath wall. These data points were fit with an exponential function to retrieve a correction factor in water [Fig. 3(a); $R^{2}$ for fit, 0.99]. The fitted exponential functions indicate increasing attenuation with increasing distance. The average correction factor for water measurements ($C_{w}$) was estimated to be $0.0009\;{\mathrm{mm}}^{-1}$.

Figures 3(d)–3(f) show acquired uncorrected NIRF maps of the reference capillary (R-) and all three target capillaries (T1-3-) acquired throughout the pullback of the target phantom when measured through water (RW, T1-3W), the 50% blood dilution (RB50, T1-3B50), and the 75% blood dilution (RB75, T1-3B75). As expected, the fluorescence intensities decreased with increasing blood content and the concomitant increase in light attenuation.

Subsequently, averaged intensity values of the reference capillary (substituting for the guidewire) recorded during target measurements at distances ranging between 0.43 and 0.53 mm in blood [RB50 and RB75, Figs. 3(b), 3(d)–3(f)] were used to quantify $C_{b}$ [see Sec. 2.5]. The average correction factors for the blood measurements were 0.0028 and $0.0034\;{\mathrm{mm}}^{-1}$ for the 50%-blood ($C_{b50\%}$) and 75%-blood ($C_{b75\%}$) dilutions, respectively [Fig. 3(c)]. These calculated values agree well (98% accuracy) with extracted correction factors ($C_{b50\%}=0.0028\;{\mathrm{mm}}^{-1}$; $C_{b75\%}=0.0033\;{\mathrm{mm}}^{-1}$) from a model fit ($R^{2}$ for fits, 0.99) applied to validation measurements of a capillary, containing the same ICG concentration as the reference, imaged in the capillary-calibration phantom at distances of 0.5 to 1.6 mm from the sheath wall through 50%-blood and 75%-blood dilutions [Fig. 3(a)]. The variance of the calculated blood correction factors over all imaging frames in the target phantom was found to be 4.8% and 2.8% for calculated $C_{b50\%}$ and $C_{b75\%}$, respectively.

Finally, we evaluated the correction accuracy of our method by applying the calculated blood correction factors ($C_{b50\%}$, and $C_{b75\%}$) to correct the NIRF signal intensities from the target capillaries, which were compared with the ground truth values measured in water. Figures 3(g)–3(i) show corrected NIRF maps of the reference capillary, and three target capillaries measured through water, 50%-blood, and 75%-blood. Because the target capillaries were angled within the phantom (see Sec. 2.6), their uncorrected NIRF intensities were acquired at distances ranging from 0.55 to 1 mm from the sheath wall to the capillary, resulting in average variances of 13% in water, 32% in 50%-blood, and 39% in 75%-blood. Furthermore, the acquired uncorrected NIRF intensities of target capillaries containing the same ICG concentration measured through 50%-blood [Fig. 3(e)] and 75%-blood [Figs. 3(f) and 3(j) did not agree with measurements in water [Figs. 3(d) and 3(j)] due to varying NIRF attenuation. In contrast, corrected NIRF intensities of all target capillaries through both 50%-blood [Fig. 3(h)] and 75%-blood [Figs. 3(i), 3(k)] show good agreement with the corrected water measurements [Figs. 3(g), 3(k)] over all acquired frames and imaging distances. In addition, we observed that the average variances of the corrected intensities over all imaging frames decreased by 10% for the water measurements, 23% for the 50%-blood measurements, and 31% for the 75%-blood measurements. Finally, Fig. 3(l) compares quantified ICG concentrations converted from corrected NIRF intensities of the blood measurements of the three target capillaries with the ground truth, which shows that our method achieved an average accuracy of 91% (average variance = 4.3%), which is a 4.5-fold improvement over uncorrected measurements.

**Fig. 4:**
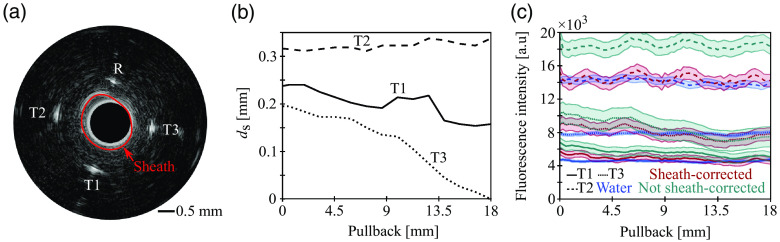
Considering catheter location inside of the sheath improves overall accuracy in attenuation correction as demonstrated with measurements through 75% blood mixture in capillary phantom. (a) Representative IVUS imaging frame showing the three target capillaries, the reference capillary, and the sheath. (b) Sensor–sheath distance ($d_{s}$) recorded by IVUS at the angular location of the three target capillaries over pullback distance. (c) Corrected intensities of the blood measurements with and without additional sheath correction and corrected intensities measured through water as the ground truth. Averaged (n=5 samples) intensities are shown as lines and standard deviation is shown as shaded areas. T1-3, target capillary 1-3; R, reference capillary.

Furthermore, we sought to quantify the impact of correcting for sensor-to-sheath distance by calculating the difference in intensity-quantification accuracy if signal loss inside of the sheath is neglected. For this analysis, we used the measurements through the 75% blood mixture. Due to natural bending of the probe, the distance between the sensor and the wall of the sheath varies at the angular locations of the different target capillaries ($d_{S_{T1-3}}$), as seen both in an exemplary IVUS frame [Fig. 4(a)] and quantified distances from an entire pullback [Fig. 4(b)]. Figure 4(c) shows corrected NIRF intensities of the target capillaries measured through blood with and without additional correction for sensor-to-sheath distance. Sheath-corrected intensities agree better with the ground truth values than the uncorrected intensities. Thus, the accuracy in ICG quantification improved by 10% for T1, 5% for T3, and 19% for T2, which provides evidence that the additional sheath correction becomes more relevant with increasing sensor-to-sheath distances [$d_{S_{T3}}<d_{S_{T1}}<d_{S_{T2}}$, see Fig. 4(b)]. Moreover, the variances of the blood-corrected intensities decreased or were unchanged when additional sheath-correction was applied (−2% for T1, 0% for T2, and −5% for T3).

## 4 Discussion

NIRF–IVUS imaging has the potential to improve assessment of coronary plaques by quantifying intravascular NIRF tracers located in the artery without the need for saline flushing; however, this necessitates the accurate correction of blood attenuation for acquired NIRF signals. Here, we demonstrated that a fluorophore-coated coronary guidewire could be employed as an NIRF reference to facilitate the accurate frame-by-frame correction of NIRF signals through blood during *ex vivo* intravascular measurements. The major advantage of this approach is that it accounts for the spatiotemporal variations of intravascular blood attenuation by enabling the estimation of frame-specific blood-attenuation correction factors, overcoming the limitations of previous methods that calculated average corrections factors.^4^^,^^9^^–^^11^

We found that it is feasible to calculate correction factors for different levels of blood attenuation from NIRF reference signals that are simultaneously acquired to tissue or phantom measurements. In an *ex vivo* porcine coronary artery perfused with 75% blood dilution, our method enabled the quantification of ICG within the arterial wall with an average accuracy of 89% compared with ground truth measurements in water. We found consistent quantification accuracy throughout the pullback (variance = 9.6%) for variable guidewire positions ranging from 0.5 to 0.85 mm [Fig. 2(c)] within an arterial lumen similar in size to human coronary arteries. Furthermore, we demonstrated with phantom capillary experiments that our method has the potential to achieve quantification accuracies as high as 91% with variances as low as 4.3% for a range of ICG concentrations (6.8, 13, and 27  μM) and for different attenuation conditions (50% and 75% blood dilutions). The higher and more consistent accuracy results from the capillary experiments compared with the tissue experiments (accuracy: 91% versus 89% and variance: 4.3% versus 9.6%) can be explained by higher variations in the corrected intensities of the guidewire compared with the reference capillary (7% versus 2.9%). Therefore, while our proof-of-concept guidewire afforded high quantification accuracy, the capillary experiments suggest that increasing the homogeneity and stability of the fluorescent coating could yield more accurate blood correction factors. Lastly, we found that the accuracy of ICG quantification was independent of the distance from the catheter sheath to the fluorescent target, as shown for distances ranging from 0.09 to 0.4 mm in the tissue and 0.55 to 1 mm in the capillary experiments. We detected and quantified ICG concentrations as low as 3.7  μM in the tissue (Fig. 2) and 13  μM in the capillary experiment (Fig. 3) through blood at maximum distances of 0.4 and 1 mm, respectively. In agreement with this finding, we validated that our correction method maintains a mean accuracy of 70% in the tissue experiments for simulated errors in IVUS distance measurements of up to 100  μm, which are expected to be smaller based on the high accuracy in measurements of known distances of the capillaries in the phantom experiments.

We used a two-media model to account for NIRF signal loss in water inside of the catheter sheath and blood attenuation between the sheath and the ICG located in the tissue or capillary. We found an improvement in ICG quantification accuracy of up to 19% for experiments in a capillary phantom when this additional sheath-correction was applied. The overall impact of the sheath-correction depends on the sensor–sheath distance ($d_{S}$), as can be observed in our results for the phantom measurements (Fig. 4). Because the location of the catheter within the sheath can vary during measurements, this additional correction could greatly improve fluorophore quantification in future intravascular NIRF imaging studies. Previous studies utilized the Twersky model for fluorescence signal correction, which is based on a two-term exponential function.^4^^,^^8^^,^^10^^,^^11^ In this study, we employ a simplified one-term exponential correction model that we validate for a range of blood attenuation levels by recording NIRF signals from a fluorophore-coated guidewire through different blood concentrations [Fig. 2(h)]. This model, contrary to two-term exponential models that introduce additional parameters, allows for a unique solution of our derived system of equations for the estimation of blood correction factors at high postprocessing speeds. Our method of correcting intravascular NIRF signals is both simple and accurate and has several advantages that could pave the way for *in vivo* studies and eventual clinical translation. First, all NIRF measurements in this study were carried out using an NIRF–IVUS catheter with dimensions appropriate for human application (see Sec. 2). Second, our method converts the coronary guidewire to an active component of intravascular NIRF–IVUS imaging, rather than a hindrance that blocks tissue signals and leaves a shadow in the data that impedes analysis. Third, we achieved high quantification accuracies using clinically approved ICG as a target fluorophore, despite ICG’s low quantum yield (2.9%^25^), in contrast to other studies that used higher quantum yield NIRF dyes such as Alexa680 (36%), Alexa750 (12%), and DiR (25%).^4^^,^^8^^,^^10^^,^^11^ Nevertheless, our method is not inherently limited to the use of ICG, and alternative fluorophores could be employed and tested in the future. Furthermore, the principle of using a guidewire as a reference standard could be translated to other intravascular fluorescence imaging modalities such as fluorescence lifetime imaging and near-infrared autofluorescence or alternative optical methods such as near-infrared spectroscopy and photo/optoacoustic in combination with a suitable coating.^3^ Even when blood is cleared from the vessel during intravascular measurements, an *in vivo* reference signal could improve standalone or hybrid imaging with IVUS or IVOCT by providing means for calibration, standardization, and quality control.

Some limitations remain to be addressed in future studies. The NIRF–IVUS measurements here were carried out at imaging speeds of 1 FPS, which was sufficient for the proof-of-concept but would likely introduce motion artifacts *in vivo*. Imaging speeds could be improved by enhancing data streaming of acquired signals or by reducing back-end vibrations and catheter friction at higher rotational speeds. We also present NIRF measurements in blood dilutions of 50% and 75% (∼20% and 30% hematocrit) instead of whole blood to account for the greater attenuation of *ex vivo* compared with *in vivo* blood.^4^ In addition, blood attenuation of NIRF signals *in vivo* might be more dynamic than the *ex vivo* perfusion used in this work. *In vivo* studies are needed to ascertain the magnitude of these effects including IVUS distance measurements under beating heart conditions. We do not anticipate significant losses in correction accuracy during *in vivo* studies based on the strategy to maintain image SNR through increased data averaging at higher imaging speeds and the presented sensitivity analysis for IVUS distance measurements. In this study, the guidewire was coated with ICG mixed with epoxy for use as a proof of concept. This modification resulted in a 25% increase in diameter on average. Improving this process by replacing parts of the standard polymer jacket of the guidewire with a thin fluorescent coating instead of overlaying it and increasing its homogeneity could reduce the footprint and yield higher quantification precision. In addition, a biocompatible fluorescent coating, such as the combination of cyanine dyes with a biocompatible polymer PMMA,^21^ must be evaluated to enable *in vivo* studies. Lastly, as in previous studies,^4^^,^^5^^,^^8^^–^^11^ our method takes into account light attenuation only by intraluminal blood flow and not by plaque tissue. This is further supported by previous studies that have indicated that the characteristic light attenuation observed in plaque tissue is much less than in blood^26^ and ICG predominantly accumulates in human plaque areas close to the vascular lumen.^6^ Furthermore, there is currently no reliable tomographic technique that can quantify the distance between the vascular wall and the fluorescent source located in deeper vascular tissue layers by NIRF–IVUS/IVOCT.

In summary, we developed a new method that utilizes a fluorophore-coated guidewire as a reference to accurately estimate blood attenuation in the form of correction factors on a frame-by-frame basis during intravascular NIRF measurements. The method facilitates high accuracy correction of signal intensities and consequent ICG quantification while having the potential for direct integration into clinical procedures.

## Data Availability

Data are available from the corresponding author upon reasonable request.
